# Corticosteroids reverse cytokine-induced block of survival and differentiation of oligodendrocyte progenitor cells from rats

**DOI:** 10.1186/1742-2094-5-39

**Published:** 2008-09-22

**Authors:** Stefan A Mann, Beatrix Versmold, Romy Marx, Sabine Stahlhofen, Irmgard D Dietzel, Rolf Heumann, Richard Berger

**Affiliations:** 1 Department of Molecular Neurobiochemistry, Ruhr University Bochum, 44780, Germany; 2 Marienhaus Klinikum Neuwied, Frauenklinik, Friedrich-Ebert-Strasse 59, 56564 Neuwied, Germany; 3 Division of Molecular Gynaeco-Oncology, Department of Gynaecology and Obstetrics, Clinical Center University of Cologne, 50931 Cologne, Germany; 4 Institute for Pathophysiology, Center for Internal Medicine, Universitätsklinikum Essen, Hufelandstraße 55, 45122, Essen, Germany; 5 Victor Chang Cardiac Research Institute, Electrophysiology and Biophysics Program, 384 Victoria Street, Darlinghurst 2095, New South Wales, Australia

## Abstract

**Background:**

Periventricular leukomalacia (PVL) is a frequent complication of preterm delivery. Proinflammatory cytokines, such as interferon-γ (IFN-γ) and tumor necrosis factor α (TNF-α) released from astrocytes and microglia activated by infection or ischemia have previously been shown to impair survival and maturation of oligodendrocyte progenitors and could thus be considered as potential factors contributing to the generation of this disease. The first goal of the present study was to investigate whether exposure of oligodendrocyte precursors to these cytokines arrests the maturation of ion currents in parallel to its effects on myelin proteins and morphological maturation. Secondly, in the search for agents, that can protect differentiating oligodendrocyte precursor cells from cytokine-induced damage we investigated effects of coapplications of corticosteroids with proinflammatory cytokines on the subsequent survival and differentiation of oligodendrocyte progenitor cells.

**Methods:**

To exclude influences from factors released from other cell types purified cultures of oligodendrocyte precursors were exposed to cytokines and/or steroids and allowed to differentiate for further 6 days in culture. Changes in membrane surface were investigated with capacitance recordings and Scanning Ion Conductance Microscopy. Na^+^- and K^+^- currents were investigated using whole cell patch clamp recordings. The expression of myelin specific proteins was investigated using western blots and the precursor cells were identified using immunostaining with A2B5 antibodies.

**Results:**

Surviving IFN-γ and TNF-α treated cells continued to maintain voltage-activated Na^+^- and K^+ ^currents characteristic for the immature cells after 6 days in differentiation medium. Corticosterone, dihydrocorticosterone and, most prominently dexamethasone, counteracted the deleterious effects of IFN-γ and TNF-α on cell survival, A2B5-immunostaining and expression of myelin basic protein. The most potent corticosteroid tested, dexamethasone, was shown to counteract cytokine effects on membrane surface extension and capacitance. Furthermore, coapplication of dexamethasone blocked the cytokine-induced downregulation of the inwardly rectifying potassium current in 80% of the precursor cells and restored the cytokine-blocked down-regulation of the voltage activated Na^+^- and K^+ ^currents during subsequent differentiation.

**Conclusion:**

Our results show that treatment of oligodendrocyte precursors with the inflammatory cytokines TNF-α and IFN-γ block the differentiation of oligodendrocyte precursors at the level of the differentiation of the voltage-gated ion currents. Co-treatment with corticosteroids at the time of cytokine application restores to a considerable extent survival and differentiation of oligodendrocytes at the level of morphological, myelin protein as well as ion current maturation suggesting the option for a functional restoration of cytokine-damaged immature oligodendrocytes.

## Background

Perinatal brain injury is frequently associated with periventricular leukomalacia (PVL), a condition characterised by disrupted myelination due to loss of oligodendrocyte progenitors [1]. In addition to hypoxic-ischemic brain damage an increased incidence of PVL in very premature fetuses has recently been related to immune responses in the brain [2-5]. For instance, in autopsies of children with PVL increased numbers of reactive interferon-γ (INF-γ) positive astrocytes have been found [6]. Studies performed in cell cultures from postnatal rat brain have shown that the inflammatory cytokines tumor necrosis factor-α (TNF-α) and IFN-γ not only induce apoptotic cell death but additionally prevent the morphological differentiation of oligodendrocyte progenitors as well as the expression of myelin-specific proteins [7-11] confirming the possible involvement of these factors in the generation of PVL.

In addition to expressing characteristic myelin proteins, such as myelin basic protein (MBP), myelin-associated glycoprotein (MAG) and 2',3' cyclic nucleotide 3'phosphodiesterase (CNP), mature oligodendrocytes differ in their pattern of ion channel expression from precursor cells. Whereas precursor cells express, among others, voltage-activated sodium and delayed K^+ ^currents these current types are down-regulated in the course of differentiation [12,13]. In differentiated oligodendrocytes the predominant current is an inwardly rectifying K^+^-current [14]. The outward rectifier K^+^-current, prominent in A2B5 progenitor cells, has been implicated in glial cell proliferation [15-18]. The inward rectifier is regarded as the prominent current involved in the removal of extracellular K^+ ^released out of active neurons and serves to maintain the negative resting membrane potential. By limiting extracellular K^+ ^activity transients it is responsible for some of the physiological functions of the mature cell (see e.g. [19]). It is so far unknown, whether the maturation block by TNF-α and IFN-γ affects the developmental down-regulation of voltage-activated ion channels as well.

Additionally, a first step in the search for a potential treatment of PVL would be to find factors that prevent the damaging effects of cytokines on the survival and differentiation of oligodendrocyte progenitor cells. Corticosteroids have been reported to exert beneficial effects in demyelinating diseases that are associated with the release of various cytokines. For instance, in acute flare-ups of multiple sclerosis, glucocorticoid treatment may reduce the severity of symptoms and speed up the recovery [20,21]. In fact, application of corticosteroids has been shown to protect mature oligodendrocytes from apoptosis induced by TNF-α and IFN-γ [22]. Since in PVL predominantly oligodendrocyte precursors are damaged we now extended these investigations by analyzing whether corticosteroids exert a protective effect on survival, protein expression, morphological and ion current maturation in cytokine-treated oligodendrocyte progenitor cells. In a preceding investigation on purified oligodendrocyte progenitors we had observed in accordance with [9] and [22], that a combination TNF-α and IFN-γ is far more potent in inducing apoptosis than either factor in separation. In the present study we thus used the cytokine concentrations previously shown to result in a survival of about 10% of the cells, containing about 35% A2B5 positive cells following six days of differentiation in culture [10].

## Methods

### Cell culture

Primary cultures of mixed glial cells were prepared from brains of 1–3-day-old Sprague-Dawley rats according to procedures described previously [23]. After decapitation the brains were transferred to a culture dish containing PBS (phosphate buffered saline: 138 mM NaCl, 8.1 mM Na_2_HPO_4_, 2.7 mM KCl, 1.47 mM KH_2_PO_4_), freed of the meninges, and mechanically disrupted by passing through a 125 μm and a 36 μm nylon mesh, respectively. After centrifugation of the cell suspension (10 min, 900 rpm at 4°C) the cell pellet was resuspended in DMEM/HAM's F12 (1:1) supplemented with 10% heat-inactivated fetal calf serum (FCS), 100 U/ml penicillin and 100 μg/ml streptomycin. Single cell suspensions were transferred to culture flasks (ca. 1.5 brains per 75-cm^2 ^flask) and preincubated at 37°C in a humidified atmosphere of air/5% CO_2_. The medium was changed first after three days in vitro and on alternate days thereafter.

After a pre-culture period for 8–11 days the cellular debris and microglia growing loosely on top of the mixed glia monolayer were removed by shaking the culture flasks for 3 h at 190 rpm. After discarding the supernatant the monolayer was washed once with phosphate buffered saline, and fresh medium was added. The phase dark cells (representing oligodendrocyte progenitors) growing on the astrocyte layer were separated by prolonged shaking at 190 rpm for 16–18 h.

The suspension was collected and centrifuged for 5 min at 1700 rpm. The pellet was resuspended (3 ml per brain) and preplated onto 10 cm^2 ^plastic dishes, to which endothelial cells, microglia and fibroblasts attached, that had been removed by the shaking procedure in addition to the oligodendrocyte precursors. After 45 min the non-adherent cells were replated onto glass coverslips coated with 0.25 mg/ml poly-D-lysin (10^5 ^cells per coverslip). After 1 h the medium was changed to proliferation medium (neurobasal medium (NB) + B27-supplement without antioxidants (both from Life Technologies GmbH, Karlsruhe, Germany) + 10 ng/ml PDGF + 10 ng/ml bFGF (both from R&D Systems, Wiesbaden, Germany)). More than 90% of the cells in culture were oligodendrocyte progenitors, as assessed by immunostaining for A2B5. Three days after plating the cells were transferred into differentiation medium (NB + B27-supplement without antioxidants + 10 ng/ml ciliary neurotrophic factor (CNTF) + 5 μm forskolin + 45 nM triiodo-L-thyronine (T3)) [24] and cultured under these conditions with a complete medium change every two days.

At 24 h after seeding parts of the cultures were treated with cytokines (recombinant rat IFN-γ (10 U/ml) and TNF-α (10 ng/ml), both R&D Systems, Wiesbaden, Germany) and/or corticosteroids (corticosterone (CS), deoxycorticosterone (DC) and dexamethasone (D), 1 μM each, Sigma Aldrich GmbH, Taufkirchen, Germany). After 48 hours treatment was terminated by transferring cells into differentiation medium containing no cytokines and/or corticosteroids. Cells of the control groups were kept under identical experimental conditions except for the application of cytokines and/or corticosteroids. Experiments were performed at day 6 after transfer into the differentiaton medium.

### Immunocytochemistry

The antibody used to characterise oligodendrocyte progenitors was the monoclonal anti-A2B5 antibody (MAB312R from Chemicon International, Hofheim, Germany). At day 9 after seeding cells growing on 12 mm coverslips were washed three times in phosphate buffered solution (PBS), fixed for 20 min in 4% paraformaldehyde in PBS and washed again three times in PBS.

Nonspecific sites were blocked in PBS containing 3% goat serum and 0.1% BSA. Then cells were exposed to the antibody solution, 1:200 in PBS/1% goat serum (Sigma Aldrich GmbH, Taufkirchen, Germany) for 1 h at room temperature. After exposure to the primary antibody, cells were washed three times with PBS and then incubated for 1 h in Alexa Fluor 488 goat anti-mouse IgM (1:1000 in corresponding buffer/1% goat serum; Molecular Probes Europe, Leiden, Netherlands). Finally cells were washed three times in PBS and the coverslips were mounted on glass microscope slides using Immuno-Fluore™ (ICN Biomedicals GmbH, Eschwege, Germany).

To assess total cell count as well as the percentage of A2B5-positive cells under various culture conditions average cell counts of 25 fields of view from each coverslip were evaluated with a 40× objective using a *Zeiss Axioplan *microscope. The total cell number was determined by counting process-bearing cells using phase contrast optics.

### Determination of cell volume and membrane surface

Cell surface and volume measurements were performed by scanning ion conductance microscopy [25,26] in the back-step mode [27,28]. Glass capillaries (thin walled GB 150-TF 8P, Science Products) where pulled in a two step process to a resistance of 6–8 MΩ when filled with bath solution, corresponding to an opening diameter of approximately 0.5–1 μm. The thin walled glass reduces both the mass of the electrode as well as unintended contacts between electrode and cell membrane at regions of the scanned cells where the slope is high. The large opening diameter additionally ensures an early detection of the approach of the capillary to the cell surface and is thus well suited for fast scans. The scanned area was 30 × 30 μm with a lateral step size of 1 μm and a vertical step size of 50 nm. Backstep size was 7 μm. With this configuration a typical scan of a cell surface took about 4 minutes, allowing scanning of several cells per cover slip. Soma volumes were calculated by summing the products of height and base area (1 μm^2^) for each point in the xy-plane with a height of at least 2 μm. Cell surfaces were determined as described in [29].

### Immunoblot analysis

At 9 days after plating cells were lysed in a buffer containing 50 mM Tris-HCl pH 7.4, 150 mM NaCl, 40 mM NaF, 5 mM EDTA, 1 mM Na_3_VO_4_, 1% (v/v) nonidet P40, 0.1% (w/v) sodium desoxycholate, 0.1% (w/v) sodium dodecyl sulfate (SDS), 1 mM phenylmethylsulfonyl fluoride (PMSF) and 10 μg/ml aprotinin. Protein concentrations in cell lysates were determined using the Bio-Rad protein assay kit.

Protein samples (10 μg per lane) were separated by sodium dodecyl sulfate (SDS)-polyacrylamide gel electrophoresis (PAGE) according to the method of Laemmli and blotted onto a nitrocellulose membrane. For detection of myelin basic protein (MBP), 2',3' cyclic nucleotide 3'phosphodiesterase (CNP) and actin we used a 12% gel under reducing conditions. Myelin-associated glycoprotein (MAG) was identified by means of a 8% gel under non-reducing conditions. Then the membrane was blocked with 5% skim milk powder in TBS (25 mM Tris-HCl, 150 mM NaCl, pH 7.5) for 1 h and incubated with suitably diluted primary antibodies for another hour (MAG 1: 10; MAB1567 Chemicon International, Hofheim, Germany/CNP 1:40; MAB326R, Chemicon International, Hofheim, Germany/MBP 1:100; SM1450, DPC Biermann, Bad Nauheim, Germany/actin 1:400; A2066, Sigma Aldrich GmbH, Taufkirchen, Germany). After three washes with TBST (TBS + Tween 20) the membrane was incubated with anti-IgG-alkaline phosphatase conjugate. The blots were finally visualized using ECL detection reagents (Amersham Biosciences Europe GmbH, Freiburg, Germany). Band intensities were analyzed by TINA 2.09 software and normalized to actin.

All experiments were performed at least three times. The numbers of coverslips analysed for each experimental condition are given in the respective figure legends. Values are given as means ± SE.

### Electrical recordings

Electrical recordings were performed in the whole cell configuration of the patch clamp technique. Prior to recording coverslips were transferred to a 3.5 cm diameter plastic Petri culture dish containing an extracellular recording solution (ExGlu) of the following composition (in mM): 110 NaCl, 5.4 KCl, 1.8 CaCl_2_, 0.8 MgCl_2_, 10 HEPES, 10 Glucose. The osmolarity was the same as that of the culture medium, 250 mOsm, the pH was adjusted to 7.3. Electrodes were pulled with a two-step puller (Narishige PP-830) from borosilicate glass tubing with filament (GB 150-TF 8P, Science Products, Hofheim) and filled with a solution containing (in mM): 100 K-gluconate, 0.1 CaCl_2_, 1.1 EGTA, 5 MgCl_2_, 10 HEPES, 3 ATP. The pH of this solution was adjusted to 7.3, the osmolarity was 235 mOsm. A liquid junction potential of this solution with respect to the bath solution of 15 mV was corrected offline. The typical resistance of recording electrodes containing this filling solution amounted to 4–5 MΩ. Recording electrodes were positioned via a 3-axis micromanipulator (M-105, Physics Instruments), which was mounted on a Zeiss IM-35 inverted microscope. Fine positioning of the electrode was controlled with a piezo actuator (DC-Mike M-232, Physics Instruments). The electrodes were connected to the headstage of an EPC7 voltage clamp amplifier (List Medical, Darmstadt, Germany). The generation of voltage protocols and the digital acquisition of currents were performed using PClamp 6 software and a Digitata 1200B AD/DA system (both Axon Instruments). Signals were filtered using the EPC7 10 kHz lowpass filter and then digitized with a sampling rate of 25 kHz. Series resistance errors were minimized by discarding recordings with access resistances of more than 20 MΩ. The formation of a high resistance seal (greater than 2 GΩ in most cases) and rupture of the membrane were monitored using small hyperpolarizing voltage pulses between 1 and 50 mV.

Na^+^-currents were monitored by a series of test pulses incrementing in 5 mV steps starting from a holding potential of -85 mV. Leakage and capacitive artifacts were subtracted using a P/4 protocol. Na^+^-current densities were determined from the peak Na^+^-current at a test potential of -20 mV normalized to the membrane capacitance calculated from the integral of the charging curve for a test potential step of 20 mV after compensation of the electrode capacitance. Voltage-activated K^+^-currents (K_v_) were determined from the average values recorded in the interval between 50–60 ms after onset of a test pulse +25 mV. To eliminate the effect of contaminating inward rectifier K^+^-currents all Na^+^- and Kv- currrents were measured in a solution containing 1 mM Ba^2+^. This treatment had no effect on the amplitudes of the Na^+ ^currents but reduced the outward currents by 47 ± 6% (n = 15) in cells after two days of cytokine treatment and by 49 ± 8% (n = 9) in control cells after 3 days in proliferation medium, corresponding a partial block of outward K^+ ^currents by Ba^2+ ^[30].

Inwardly rectifying K^+ ^currents (K_IR_) were determined applying a test pulse protocol consisting of a series of test pulses from -175 mV to +20 mV in the absence of automatic leakage and capacitive current subtraction. K_IR _currents were determined as the Ba^2+^-blockabale part of the current by applying the same voltage protocol in ExGlu solution first followed by a switch to ExGlu solution supplemented by 1 mM BaCl_2_, and subtracting the recorded traces for each voltage step. The current density was determined from the difference trace at a test voltage of -135 mV normalized to the cell capacitance calculated from the integral of the charging curve. A cell was regarded as expressing a K_IR _current when the current exceeded 50 pA. A simple gravity controlled perfusion system was used to apply either ExGlu or K_IR_-blocking solution through a multibarrel pipette. The perfusion was adjusted initially by monitoring the flow of an ink solution to asssure a complete solution exchange after switching between the two supplies. The liquid level was controlled via an adjustable suction needle, connected to a reversed "Optimal" aquarium pump (Schego, Offenbach, Germany). Cells for surface scanning or electrical recording were selected under phase contrast optics. On day 3 in culture, irrespective of treatment conditions, most cells showed a bipolar morphology and these were selected for recording. One aim of this investigation was to find out, whether cells retaining the bipolar morphology and A2B5 positivity after cytokine treatment followed by 6 days in differentiation medium also maintain the immature ion current pattern or whether voltage-activated cation currents are down-regulated by the incubation in differentiation medium irrespective of the maintenance of the immature morphology of the cells. To this aim recordings on day 9 after seeding in cultures treated with cytokines only were performed on bipolar cells. To test, whether morphologically differentiated cells show a downregulation of voltage-activated Na^+ ^and K^+ ^currents, as expected during normal differentiation of oligodendrocytes, multipolar cells were investigated on day 9 in the three other populations showing more than 90% multipolar cells staining negative for A2B5.

The statistical significance of differences between groups was assessed by ANOVA followed by Tuckey's Post-hoc Test. All data represent mean value ± SE.

## Results

**Figure 1:**
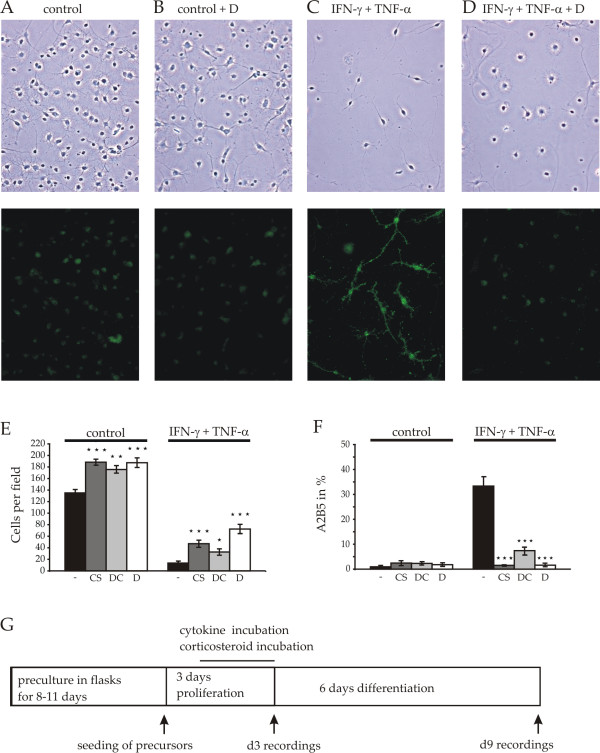
**survival and A2B5 staining of oligodendrocytes following treatments with cytokines and corticosteroids**. Phase contrast microphotographs as well as anti-A2B5 staining after three days in proliferation medium followed by 6 days in differentiation medium. Note, that after treatment of oligodendrocyte precursors with TNFα (10 ng/ml) and IFNγ (10 U/ml) for 48 h on 2–3 days *in vitro *(C) cells display mostly bipolar morphologies typical for progenitor cells, maintaining their A2B5 immunopositivity. Coapplication of dexamethasone during cytokine treatment (D) in the proliferation medium restored the extension of multipolar arborizations and the downregulation of A2B5 staining. (E) Average number of cells per field of view at culture day 9 (for every bar 25 fields of view from 12 coverslips of the control cultures and 4 coverslips of the corticosteroid treated cultures were counted). In both, control (left bars) and in cytokine-treated cells (right bars) corticosteroids caused a significant increase in the total number of cells surviving in culture. (F) Percentage of A2B5 positive cells at culture day 9. Note, that in control cells not treated with cytokines (left four bars) no significant effect of corticosteroids on the number of A2B5-positive cells was detected, whereas in cytokine-treated cultures the percentage of A2B5-positive cells was decreased by cotreatment with corticosteroids (CS: corticosterone, DC: deoxycorticosterone, D: dexamethasone, no corticosteroid treatment (-) * p < 0.05, ** p < 0.01, *** p < 0.001. For reasons of comparison the black bars in E and F are based on data published in [10], Figure 3B. Since cell counting was performed directly at the fluorescence microscope, fields of view were slightly larger than the photomicrographic frames shown. (G) Flow chart of the experimental design: After day 1 in proliferation medium, test cultures of oligodendrocyte precursors were treated for 48 hours with control proliferation medium or 10 U/ml IFN-γ and 10 ng/ml TNF-α in the presence or absence of glucocorticoids. The treatment was stopped by transferring the cells into differentiation medium. To judge long-term effects of treatments of precursor cells on subsequent differentiation most investigations were performed following 6 days in differentiation medium.

The effect of a transient treatment of progenitor cells with cytokines and the effect of a coapplication of corticosteroids on the subsequent differentiation into mature oligodendrocytes were studied using the culture protocol depicted in Figure 1G. Total cell numbers, percentages of A2B5 immunopositive cells, soma and process volumes and surface areas, membrane capacitances, the expression of myelin specific proteins and voltage activated Na^+^, K^+ ^as well as inwardly rectifying K^+ ^currents were evaluated following six days of differentiation in culture.

### Corticosteroids attenuate cytokine induced cell death and decrease the percentage of A2B5-positive cells

To extend previous qualitative observations suggesting that corticosteroid treatment counteracts apoptosis and arrest of maturation [31] isolated progenitors were treated for 3 days in proliferation medium, transferred into differentiation medium and investigated six days later (Figure 1G). We had previously shown that incubation with IFN-γ and TNF-α caused a dramatic loss in numbers of surviving cells as compared with controls [10]. We now repeated these experiments in the presence of glucocorticoids. After co-application of cytokines and glucocorticoids the number of surviving cells per field amounted to 32.7 ± 4.9 (DC, deoxycorticosterone), 47.1 ± 5.4 (CS, corticosterone) and 72.5 ± 7.4 (D, dexamethasone, 25 fields of view on 4 coverslips counted for each condition) which was significantly higher than the 13.7 ± 1.9 cells previously found to survive in control cultures. Control cultures treated with corticosteroids in the absence of cytokines showed a slight 0.4-fold increase in the total cell number (188.3 ± 4.6 (CS), 175.7 ± 5.6 (DC) and 187.4 ± 7.6 (D), vs. 135.0 ± 5.8 n = 12, Figure 1E) compared with the previously observed number of 135 ± 5.8 for control cultures [10].

**Figure 2:**
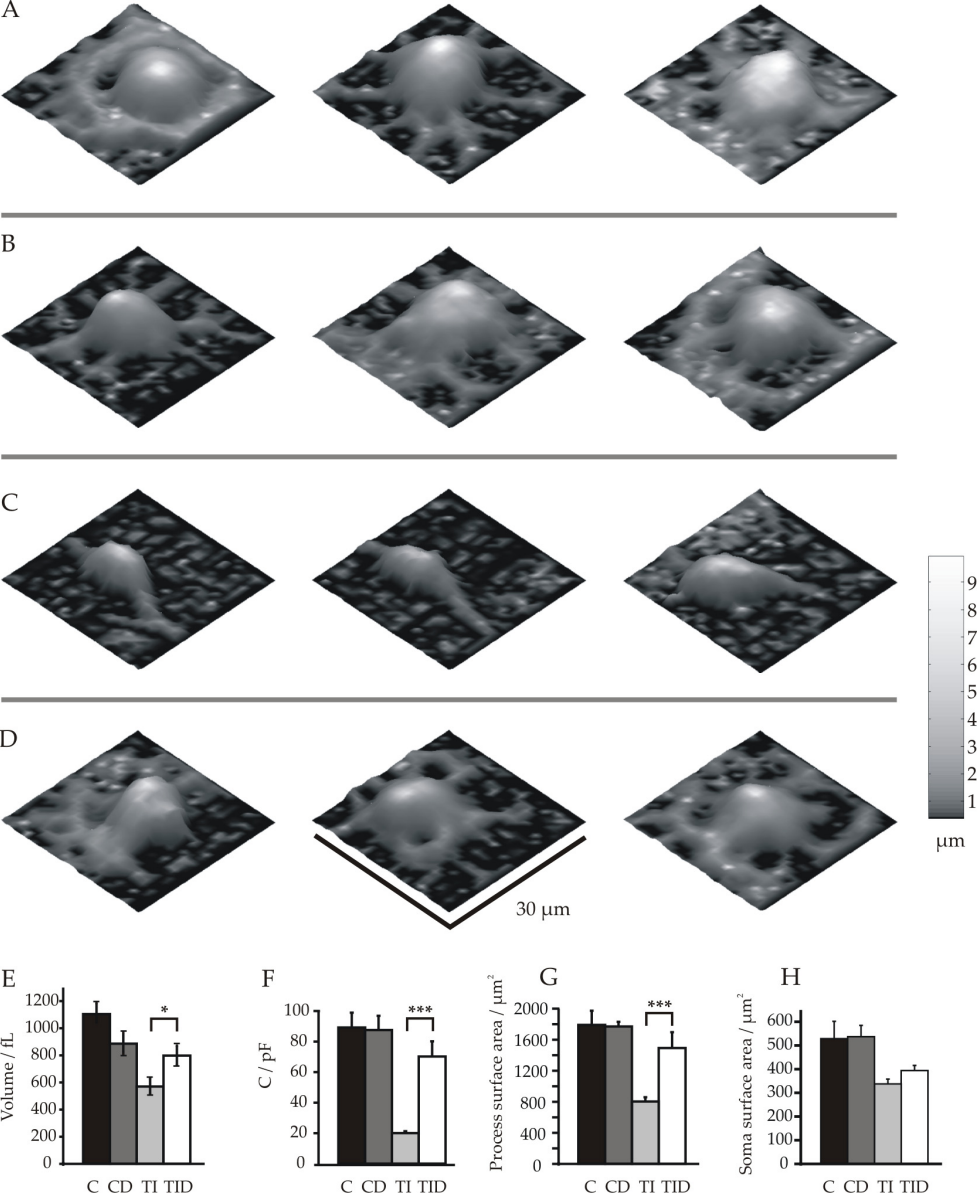
**Morphological differences of cells cultured for 6 days in differentiation medium after various treatments visualized by Scanning Ion Conductance Microscopy (SICM)**. (A) Samples of control oligodendrocytes pretreated with proliferation medium alone, (B) proliferation medium supplemented with dexamethasone, (C) proliferation medium supplemented with IFN-γ and TNF-α, (D) proliferation medium supplemented with IFN-γ, TNF-α and dexamethasone. Scans were performed in a frame of 30 × 30 μm with 1 μm lateral step size. Calibration bar converting height into greyscale is shown at the right. (E) Average volume measurements of cell somata (height > 3 μm), calculated from the SICM-data. Note, that the smallest volumes correspond to cells pretreated with TNF-α and IFN-γ only and the reversal by cotreatment with dexamethasone. (F) Capacitances of cells cultured under the same conditions as cells shown in A-D analysed using the whole cell voltage clamp technique. (G) Surface of cell processes with a height of more than 0.5 μm above the culture dish, calculated from the SICM data. (H) Soma surface (height of more than 3.0 μm above the culture dish) calculated from the SICM data. Note, that the TNF-α and IFN-γ treatment and dexamethasone rescue affect predominantly the process surface. Hence most of the capacitance is contributed by the process membrane. Error bars indicate mean ± SE, * p < 0.05, ** p < 0.01, *** p < 0.001. C: control, DC: treatment with dexamethasone, TI: treatment with TNF-α and IFN-γ, TID: cotreatment with TNF-α and IFN-γ plus dexamethasone each for two days in proliferation medium

We had previously observed, that in differentiating control cultures the percentage of A2B5 positive cells declined after 6 days in differentiation medium to 1.0% and the cells developed the characteristic arborisation of mature oligodendrocytes (see e.g. Figure 2A). Likewise, we now observed that this fraction is comparable after treatment of control cultures with one of the three corticosteroids yielding a similar reduction of A2B5-positive cells, amounting to 2.5 ± 0.8% (CS), 2.3 ± 0.5% (DC), 1.8 ± 0.5% (D), n = 3 coverslips evaluated for each corticosteroid (Figure 1F). In contrast to this down-regulation of A2B5 positive cells we had observed that 36% of the IFN-γ/TNF-α-treated surviving cells still stained positive for A2B5 (Figure 1C, F) after six days in differentiation medium and the cells maintained a bipolar morphology (Figure 1C, Figure 2C) as compared with the extensive arborizations developed by the differentiated control cells (Figure. 1A, 2A). Remarkably, we now observed that the cytokine-induced preservation of the A2B5 phenotype was almost completely reversed by coapplication of corticosteroids. While after cytokine treatment 36.3 ± 3.9% (n = 9) of the cells had maintained the A2B5 antigen expression [10] the percentage of A2B5-positive cells reversed to values found in control cultures after cotreatment with corticosteroids (1.5 ± 0.1% (IFN-γ + TNF-α + CS); 7.4 ± 1.4% (IFN-γ + TNF-α + DC); 1.6 ± 0.4% (IFN-γ + TNF-α + D, Figure 1F, n = 3 coverslips evaluated for every corticosteroid).

### Restoration of membrane growth during differentiation of dexamethasone protected cells

Cytokine and corticosteroid effects on the surface membrane extension of the cells were investigated using Scanning Ion Conductance Microscopy (SICM) and capacitance measurements in the whole cell configuration of the patch-clamp technique. Differentiated control cells showed an average membrane capacity of 89 ± 9 pF (n = 16), a soma volume of 1107 ± 81 fL (n = 10), an average soma surface of 539 ± 72 μm^2 ^(n = 8) as well as an average cell surface of 1793 ± 180 μm^2 ^(n = 8) in the scanning frame of 30 μm × 30 μm.

Cytokine treated cells showed, however, significantly smaller membrane capacitances than control cells (20 ± 1 pF, n = 29; p < 0.001) indicating 4.5 times less membrane surface per cell (Figure 2F). This was further confirmed with SICM-measurements showing significantly smaller soma volumes of 572 ± 60 fL, (n = 8, p < 0.05) compared with control cells (Figure 2E). The soma surfaces were 339 ± 19 μm^2 ^(n = 9, p = 0.047) and reduced to a smaller extend (a factor of 1.6) than the whole cell surfaces (806 ± 52 μm^2^, n = 9, p < 0.001), which were reduced by a factor of 2.2 (Figure 2G and 2H). The smaller reduction of the soma surface compared with the cell surface quantitatively confirms the intuitive impression that the cytokine treated, more immature cells produce a smaller myelin generating membrane surface (Figure 2C).

Cytokine-treated cells were then cotreated with dexamethasone, the steroid that had shown the most potent effect on cell survival (Figure 1E). Dexamethasone-treatment had no effect on cell capacitances, surface areas and volumes of control cells (Figure 2B, E–H). However, co-treatment of cytokine-exposed cells with dexamethasone led to significantly larger cell sizes (Figure 2D). Hence, cotreatment with dexamethasone increased the average soma volume by a factor of 1.4 to 800 ± 72 fL (n = 11, Figure 2E). Whereas the average soma area was only insignificantly larger (395 ± 21 μm^2^, n = 10) than the soma area of the cytokine-treated cells (339 ± 19 μm^2^, n = 9) after dexamethasone cotreatment, this treatment led to a significant increase of the area of the cell processes by a factor of 1.9 to 1495 ± 202 μm^2 ^(n = 7, p < 0.01, Figure 2G and 2H). This finding is further supported by an even larger increase of the membrane capacitance from 20 ± 1 pF (n = 29) to 70 ± 10 pF (n = 15; Figure 2F).

### Cytokine-induced decreases in protein levels of MAG, MBP and CNP are attenuated by glucocorticoids

**Figure 3:**
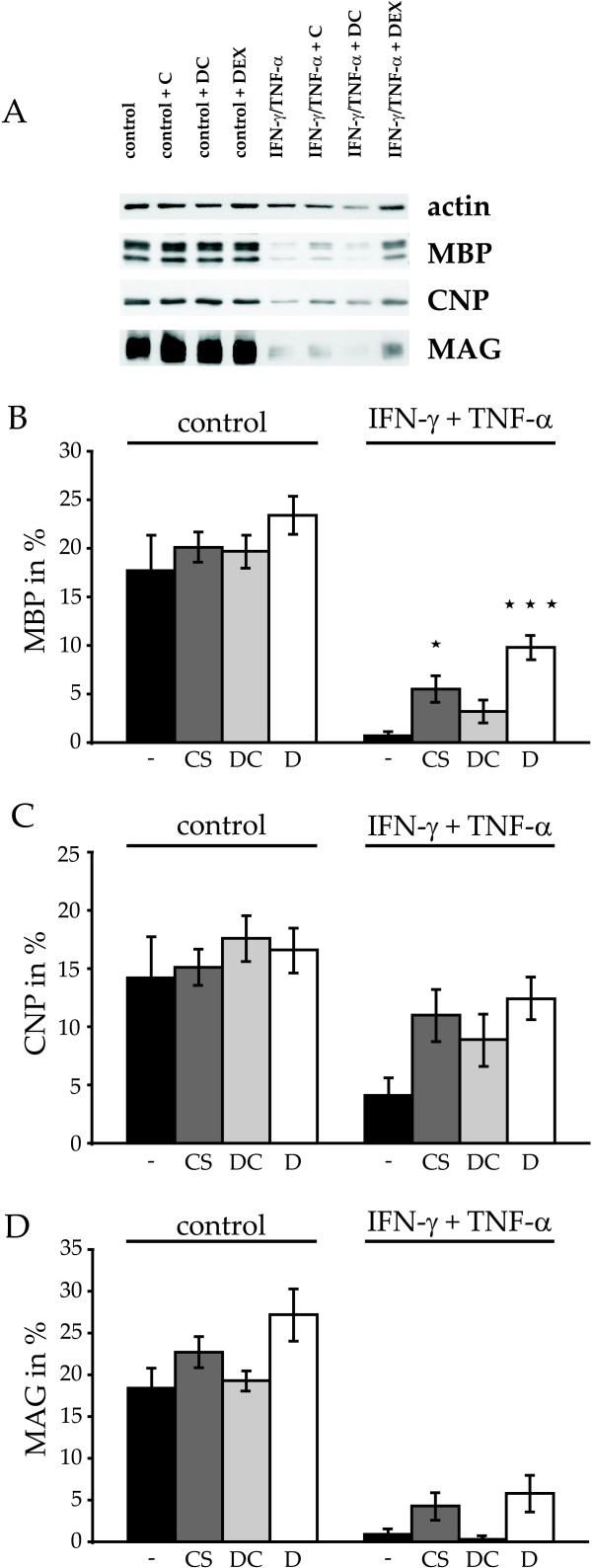
**Protein expression of MAG, MBP and CNP at culture day 9**. Note the strong reductions of myelin protein expression normalized to actin in western-blots from cells preincubated with IFN-γ and TNF-α (bars to the right). Cotreatment with corticosterone (CS), deoxycorticosterone (DC) or dexamethasone (D), significantly attenuated the inhibition of protein expression for MBP (B) and showed a non-significant tendency for a recovery of CNP (C) and MAG (D), *p < 0.05, ***p < 0.001. For reasons of comparison data shown in (B) are based on the same dataset as those shown in Figure 2A of [31].

We next tested the glucocorticoid effects on characteristic proteins involved in myelination. Similar to the morphological parameters the levels of MAG, MBP and CNP proteins were not influenced by the glucocorticoids tested (Figure 3). In accordance with previous observations [10,31] protein levels of MBP, MAG, and CNP were, however, significantly reduced by cytokines from 17.7 ± 3.5%, 18.4 ± 2.8% and 14.4 ± 3.5% to 0.7 ± 0.3%, 0.9 ± 0.5% and 4.1 ± 1.4% (Figure 3, n = 6 coverslips for MBP & CNP, n = 3 coverslips for MAG). Coapplication of corticosterone and dexamethasone significantly reversed the decrease in protein expression of MBP to 5.5 ± 1.3% and 9.8 ± 1.2% (Figure 3, n = 6 coverslips for each condition). A non significant tendency for an increased expression of CNP and MAG was additionally observed (Figure 3), suggesting that the restoration of the expression of myelin proteins by dexamethasone may be extended to further membrane proteins.

### Cytokine and dexamethasone action on voltage-activated sodium currents

**Figure 4:**
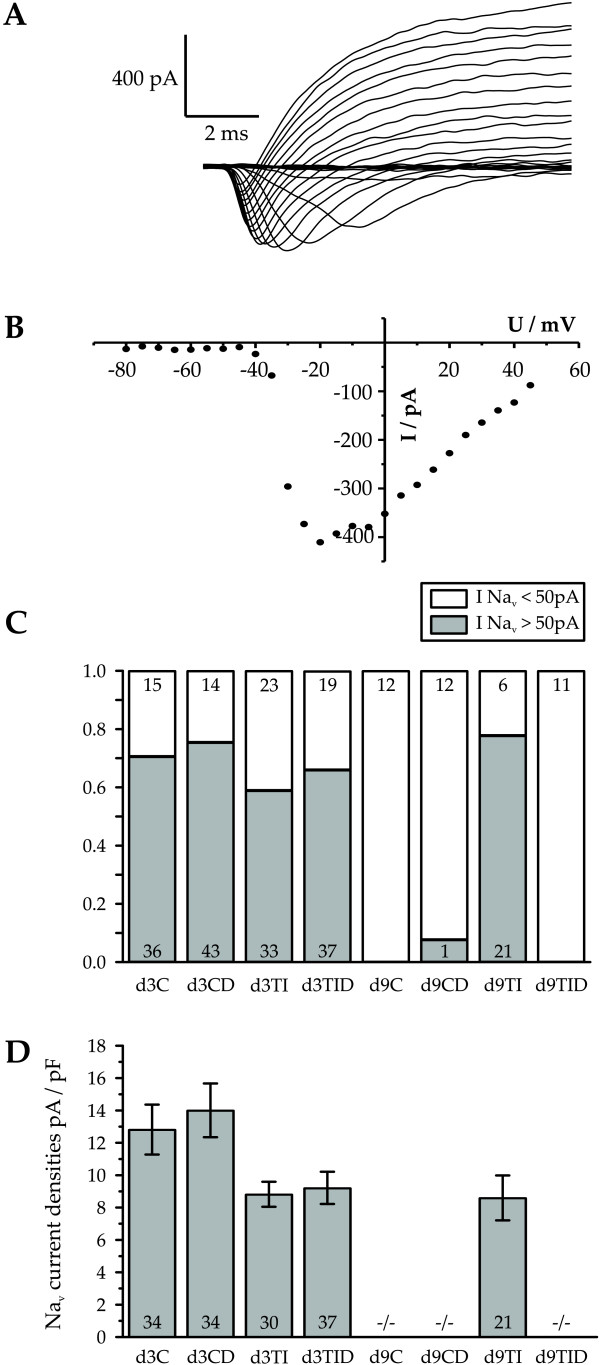
**Voltage-activated sodium currents after three days in proliferation medium and further six days in differentiation medium**. (A) Original current traces from cell cultured for three days under control conditions recorded in Ba^2+ ^containing extracellular solution to avoid distortion of voltage-activated currents by inwardly rectifying potassium currents. (B) Current to voltage relationship of peak sodium currents shown in A. (C) Fraction of cells showing a I_Na _current larger than 50 pA for each tested culture condition (control: C, pretreatment with TNF-α and IFN-γ for 48 hours:TI, control cultures treated with dexamethasone: CD, cultures co-treated with dexamethasone and TNF-α and IFN-γ for 48 hours: TID). The number of cells tested under each condition is indicated within the corresponding column. (D) Average inward current densities of the cells listed posititve for this current in (C). Note, that d9TI cells show a similar percentage of cells with inward currents of similar amplitudes as proliferating progenitor cells. The single cell showing an inward current under condition d9CD was ignored for the analysis of the average current density. The sodium current densities were not statistically significantly different from each other. Error bars denote mean ± SE.

In accordance with previous publications reporting voltage-activated Na^+ ^currents in oligodendrocyte precursor cells 70% of the bipolar cells investigated after 3 days in proliferation medium showed voltage-activated sodium currents of more than 50 pA (Figure 4C, d3C), displaying an average Na^+^-current density of 12.8 ± 1.5 pA/pF (n = 34, mean ± SE, Figure 4D). In control cultures treated with dexamethasone we observed Na^+ ^currents larger than 50 pA in a slightly larger population of 75% of the cells showing a slightly larger current density of 14.0 ± 1.7 pA/pF (n = 34). Oligodendrocyte precursor cell cultures treated for two days with IFN-γ + TNF-α showed a reduced percentage of 58% of cells expressing Na^+^-currents with insignificantly reduced (p > 0.05) amplitudes of 8.8 ± 0.8 pA/pF (n = 30, Figure 4C, D d3TI). Again, cotreatment with dexamethasone led to a slight increase in the number of cells expressing Na^+ ^currents to 66% showing a slight, but insignificantly increased Na^+ ^current amplitude of 9.2 ± 1.0 pA/pF (n = 37, Figure 4C, D d3TID). Following further six days in differentiation medium the cells had changed their morphological appearance to multipolar cells. In none of the 12 cells from control cultures investigated a voltage-activated Na^+ ^current was found (Figure 4C, d9C). Likewise, in the dexamthasone-treated control cultures only one cell out of 13 cells investigated showed a Na^+^-current. In contrast, 77% of the cytokine treated cells that had been cultured in differentiation medium for 6 days and which had maintained a bipolar morphology still showed voltage – activated Na^+ ^currents with an average amplitude of 8.6 ± 1.5 pA/pF (n = 21, Figure 4C and 4D, d9TI). To investigate whether dexamethasone affects the arrest of sodium current downregulation by TNF-α and INF-γ, Na^+^-currents were studied in cytokine and dexamethasone co-treated cells followed by further differentiation for 6 days. As depicted in Figure 4C, D following co-treatment with cytokines and dexamethasone none of the 11 cells investigated continued to show voltage-gated Na^+^-currents (Figure 4C – d9TID).

Hence, in addition to preventing morphological maturation treatment with TNF-α and INF-γ also prevents the downregulation of voltage-activated sodium currents that occurs during normal maturation of oligodendrocytes. This effect can be reversed by cotreatment with dexamethasone.

### Cytokine and dexamethasone effects on voltage activated potassium currents

**Figure 5:**
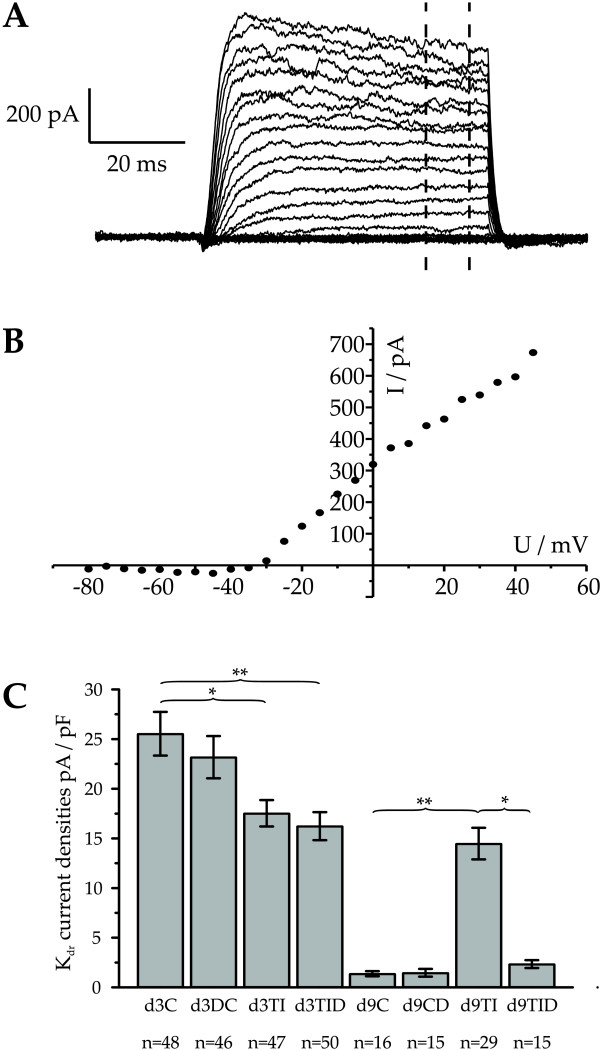
**Outward rectifying currents after three days in proliferation medium and further six days in differentiation medium**. (A) Original current traces in an extracellular solution containing 1 mM Ba^2+ ^to eliminate contributions of inwardly rectifying K^+ ^currents from cell cultured under condition d3C. (B) Current to voltage relationship of mean currents shown in (A) measured between 45 and 55 ms between the two vertical dashed lines in (A). (C) All cells recorded from showed delayed rectifier currents (I_KDR_) (control: C, pretreatment with TNF-α and IFN-γ for 48 hours:TI, control cultures treated with dexamethasone: CD, cultures co-treated with dexamethasone and TNF-α and IFN-γ for 48 hours: TID, numbers of cells investigated given below bars). Note, that cytokine treatment significantly reduced I_KDR _after 3 days in proliferation medium in accordance with [52]. In contrast to control cells, in which a significant down-regulation took place after 6 days in differentiation medium (d3C versus d9C), outward K^+^-currents in cytokine pretreated biopolar cells showed no further down-regulation in differentiation medium (d3TI versus d9TI, p > 0.05). A coincubation with dexamethasone however, led to a signficant downregulation of I_KDR _in the surviving multipolar cells (d9TI versus d9TID) resulting in a I_KDR _density indiscernable from that recorded in cells differentiated under control conditions (d9TID versus d9C, p > 0.5). Error bars denote mean ± SE. * p < 0.05, ** p < 0.01, *** p < 0.001).

During maturation of oligodendrocytes voltage-activated K^+^-currents have been shown to be down-regulated in parallel with voltage-activated Na^+^-currents. In the following we thus investigated whether cytokine treatment and co-treatment with dexamethasone affects K^+^-currents as well. All precursor cells maintained for three days in proliferation medium showed delayed K^+^-outward currents averaging 25.5 ± 2.2 pA/pF (n = 48, Figure 5C, d3C), consistent with previous reports on oligodendrocyte precursor cells in PDGF-containing medium [16]. Following addition of dexamethasone for two days to control proliferation medium insignificantly smaller K^+ ^currents of 23.2 ± 2.1 pA/pF (n = 46) were observed. In oligodendrocyte precursor cells treated for three days with IFN-γ + TNF-α the average amplitudes of the voltage-activated K^+ ^currents were significantly reduced to 17.5 ± 0.4 pA/pF (n = 47, Figure 5C, d3TI). Again, the coincubation with dexamethasone had only a slight effect and resulted in outward K^+ ^current amplitudes of 16.2 ± 1.4 pA/pF (n = 50).

As expected for the differentiation of oligodendrocytes after 6 days in differentation medium control cells showing multiple arborizations only showed small residual delayed K^+ ^currents of 1.4 ± 0.3 pA/pF (n = 16, Figure 5C – d9C). Likewise, current densities in dexamethasone-treated control cells were reduced by a similar extent (Figure 5C d9CD).

However, bipolar cells maintained for 6 days in differentiation medium after cytokine treatment continued to express delayed rectifier K^+ ^currents of on the average 14.5 ± 1.8 pA/pF (n = 29, Figure 5C, d9TI), which is in accordance with the maintenance of the undifferentiated phenotype, as well as the Na^+ ^current expression of these cells. Following co-treatment of the proliferating precursor cells with cytokines and dexamethasone the delayed rectifyer K^+ ^current density was reduced on the average to the level found in differentiated cells (2.4 ± 0.5 pA/pF, n = 15 – Figure 5C- d9TID). In accordance with our findings concerning sodium currents, these observations suggest, that cytokine pre- treatment prevents normal differentiation of potassium outward currents and that the cytokine effects can be prevented by co-treatment with dexamethasone.

### Inwardly rectifying K^+ ^currents

**Figure 6:**
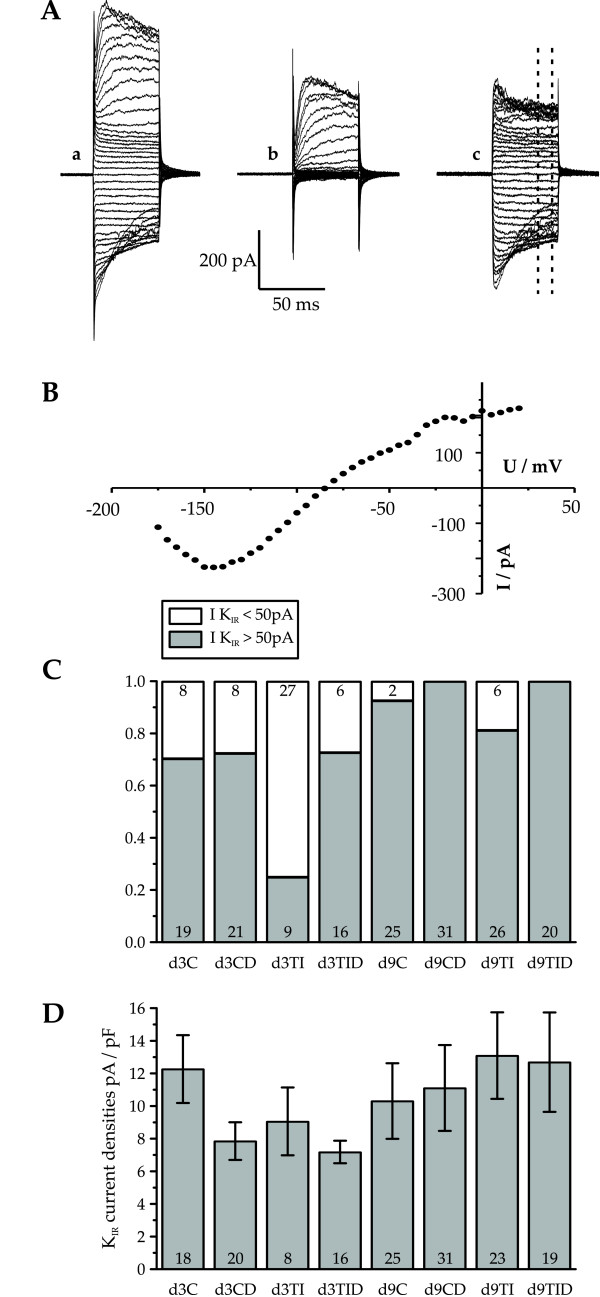
**Inwardly rectifying K^+ ^currents after three days in proliferation medium and further six days in differentiation medium**. (A) Original voltage clamp recordings of current traces from cell cultured after 9 days in control solution. No leak and capacitance subtraction used to visualize current components at a holding potential of -85 mV. (Aa) Current traces in physiological extracellular solution, (Ab) in the same solution containing in addition 1 mM Ba^2+^. (Ac) plot of the difference between traces shown in (Aa) and (Ab) corresponding to the inwardly rectifying K^+ ^current (K_IR_). (B) Current versus voltage relationship for recordings shown in Ac (mean current between the dashed lines in Ac). (C) Fraction of cells showing a K_IR _current larger than 50 pA for each tested culture condition. The number of cells tested under each condition (control: C, pretreatment with TNF-α and IFN-γ for 48 hours:TI, control cultures treated with dexamethasone: CD, cultures co-treated with dexamethasone and TNF-α and IFN-γ for 48 hours: TID) is indicated within the corresponding column. (D) Average current densities of K_IR _for the cells listed positive for this current in (C). Note, that after 3 days in proliferation medium containing TNF-α and IFN-γ for 48 hrs the number of cells showing a K_IR _was reduced to only 20%. Error bars indicate ± SE.

After three days in proliferation medium 70% of the control cells investigated showed an inwardly rectifying K^+ ^current exceeding 50 pA (mean value 12.3 ± 2.1 nA/pF – Figure 6C, D – d3C). Most of the inwardly rectifying K^+ ^currents showed an inactivation at negative membrane potentials reflected in the negative slope of the I/V relationship (Figure 6Aa, B). We did not further examine whether this inactivation was voltage-dependent [32] or due to the presence of Na^+ ^in the recording solution [30]. However, we only observed the inactivation in about 60% of the cells recorded from using identical solutions and voltage protocols, suggesting an inhomogeneous expression pattern in the oligodendrocytes. Following treatment with dexamethasone a similar fraction of 72% of the cells recorded from showed inwardly rectifying K^+^-currents, with an insignificantly reduced current density of 7.9 ± 0.5 pA/pF (n = 20, Figure 6C, D – d3CD). Remarkably, in oligodendrocyte precursor cells treated for two days with IFN-γ + TNF-α only 20% of the cells investigated showed an inwardly rectifying K^+ ^current exceeding 50 pA (Figure 6 – d3TI). The average current density of the 9 cells out of 45 cells recorded, that showed a current exceeding 50 pA displayed a slightly, but insignificantly (p > 0.05) smaller amplitude as recorded in the control cells (9.1 ± 2.1 pA/pF). Most interestingly, co-treatment with dexamethasone restored the percentage of cells expressing inwardly rectifying potassium currents to 72%, showing almost identical current densities as observed in only dexamethasone treated control cultures (7.2 ± 0.7 pA/pF, n = 16).

Following 6 days in differentation medium in control cultures the inwardly rectifying K^+ ^current was the dominant current. While the absolute value of the K_IR _current, which was expressed in 92% of the cells investigated had increased from 169 ± 29 pA in the control percursor cells to 864 ± 190 pA in the morphologically differentiated cells the average current density, amounting to 10.3 ± 2.3 pA/pF (n = 25, Figure 6 – d9C) had only changed insignificantly. Similarly, after 6 days in differentiation medium 81% of the cytokine-treated cells expressed inwardly rectifying K^+ ^currents of an average density of 13.1 ± 2.7 pA/pF (n = 27, Figure 6C, D – d9TI). Due to the smaller size of the cells, the absolute current amplitudes (210.4 ± 40.6 pA/pF, n = 27) were only slightly larger than those of the cytokine treated precursor cells at d3 (155.7 ± 35.7 pA/pF). Remarkably, following 6 days after removal of cytokine treatment and exposure to the differentiation medium, K_IR _was recorded again in more than 80% of the cytokine treated cells. This was slightly less than in the dexamethasone pretreated and control differentiated cells (Figure 6C, D – d9CD and d9TID), which expressed K_IR _in 100% of the cells recorded from.

## Discussion

### Cytokine effects on differentiation of oligodendrocyte precursors

Here we present the novel observations, that oligodendrocyte progenitors surviving inflammatory cytokine treatment maintain the voltage – activated Na^+^- and K^+^-currents characteristic of oligodendrocyte progenitors [12,13,33]. Using a recently developed scanning ion conductance microscope [29] we quantitatively determined that the cytokine treatment impairs the extension of the surface of the processes to a larger extent than the soma surface. Hence, in accordance with the observed reduction in the relative expression of MBP, CNP and MAG, cytokine-treated progenitor cells develop less membrane surface to accommodate myelin proteins during subsequent differentiation time in culture. The cytokine-treatment thus seems to arrest the developmental program inducing the expression of several features of differentiated oligodendrocytes as a set in bipolar surviving cells. We have previously observed (Figure 3A in [10]) that the higher percentage of A2B5 positive cells in surviving cytokine treated cells also corresponds to a larger absolute number of A2B5 positive cells and can thus not be easily explained by a preferential survival of immature cells. The cytokine treatment conditions used in the present study did not affect the cell density within the first three days in culture [10]. Hence, the larger percentage of immature cells surviving after 6 days in differentiation medium could not be attributed to a lower cell density at the starting point of differentiation. Possible contributions of the decreasing cell density during the following days on the arrest of differentiation, caused by a possible reduction of paracrinely released survival factors other than those supplied in the medium, e.g. endocannabinoids [34] remain to be investigated in more detail.

### Rescue of cytokine-treated cells by corticosteroids

Furthermore, our experiments demonstrate, that coincubation of cytokines with corticosteroids increases the percentage of surviving oligodendrocytes, reduces the percentage of A2B5 positive cells at day 9 to 2% and restores myelin protein expression and expansion of membrane surface. In addition the rescued multipolar cells show a down-regulation of voltage activated Na^+^- and delayed K^+ ^current densities characteristic for differentiated oligodendrocytes. Since the absolute values of the voltage-activated Na^+^- and outward K^+ ^currents were increased in the cytokine treated cells compared with control and dexamethasone treated cells at day 9 in culture (data not shown) the down-regulation of the current densities in the course of differentiation cannot be attributed to a simple dilution of preexisting channel proteins in the increased membrane surface. Taken together this suggests that corticosteroid treatment counteracts the cytokine-induced inhibition of maturation.

Our cell counts confirm observations of [22], who showed a protection of more mature oligodendrocytes, than used in the present investigation, against cytokine-induced cell death by corticosteroids. Whereas in the present study dexamethasone, binding to glucocorticoid (type II) receptors was clearly more potent, aldosterone, binding like deoxycorticosterone, to mineralocorticoid (type I) receptors, was similarly effective in this study, suggesting the involvement of both types of glucocorticoid receptors in the protective mechanism.

### Corticosteroid effects on apoptosis, myelin proteins and differentiation

Corticosteroids have been reported to promote oligodendrocyte maturation in various aspects. Barres et al. [35] reported, that 1 μM dexamethasone slowed the average cell-cycle time and allowed differentiation of GC-positive oligodendrocytes, thus inhibiting progenitor proliferation (see also [36]). De Vellis and colleagues [37,38] found, that glycerol phosphate dehydrogenase, an oligodendrocyte specific enzyme involved in membrane lipid biosynthesis, is inducible by glucocorticoids. Likewise, Poduslo et al. [39] reported that addition of hydrocortisone to oligodendroglia in culture increases the level of mRNA for proteolipid protein, increases the synthesis of cerebrosides, and activates ketone body metabolizing enzymes, which serve as precursors for lipid synthesis during development. Ved et al. and Byravan and Campagnoni [40,41] additionally observed an increase in MBP synthesis by hydrocortisone after more than 15 days in mixed brain cultures from mice. In one study, in contrast, dihydrodeoxycorticosterone has been reported to decrease mRNA levels of MBP, whereas other corticoids did not cause any effects on the gene expression of this protein [42]. This could be explained by experiments indicating a maturation dependent effect of dexamethasone. Thus dexamethasone had a stimulatory effect on myelin proteins which later in culture reversed to an inhibitory effect [43]. In other glial cell types, including glioma and astrocytoma cell lines [44] as well as in fibroblasts [45] and hepatoma cells [46] similar anti-apoptotic effects of glucocorticoids have been noted. However, the antiapoptotic and differentiation promoting effect of dexamethasone is not universal. In many other cell types, for example thymocytes and some leukemia cell lines, treatment with glucocorticoids induces apoptosis [47], which has led to their common use as chemotherapeutic agent in lymphomas and leukemias [48].

### Corticosteroid effects on voltage-activated ion currents in oligodendrocytes

This is to our knowledge the first investigation of dexamethasone actions on voltage-activated ion currents in oligodendrocyte precursor cells. We observed, that two days of dexamethasone treatment induced no changes of outward K^+^- and voltage-activated Na^+^-inward current density in control or cytokine treated cells. In other cell types, such as pituitary GH3 cells, dexamethasone has been shown to cause an enhanced transcription of the Kv1.5 gene as well as effects on the open probability of calcium-activated BK channels. In T- lymphocytes, in contrast, a down-regulation of the expression of the Kv1.3 subunits has been observed [49-51]. The divergent effects on K^+^-channels in different cell types correspond to the cell type-specific effects of corticosteroids on survival and apoptosis discussed above, suggesting that cytokines as well as corticosteroids trigger concerted programmes in specific cells, the details of which are just starting to be understood.

### Corticosteroid effects on K^+ ^inward currents

Earlier investigations on mature oligodendrocytes from 4–6 months old lambs showed decreases in both, K^+^-inward as well as outward currents following 48 hrs incubation of 2-week-old cultures with TNF-α [52]. In accordance with these investigations, McLarnon et al. [53] found diminished mean open times of inwardly rectifying K^+ ^channels in TNF-α preincubated cultured human oligodendrocytes. Our finding of inwardly rectifying K^+^-currents of less than 50 pA in 80% of the cells investigated extends these observations to rat oligodendrocyte precursor cells. This downregulation was only observed in cells investigated shortly after the cytokine incubation. In cells investigated 6 days later inward rectifying currents were almost restored. The cytokine effect may even decline with a time constant in the range of hours, since in the present series of experiments inward currents showing current densities > 50 pA were never observed in the first 20 minutes of a recording session. Most interestingly, in cultures co-treated with dexamethasone the percentage of cells showing inwardly rectifying K^+ ^currents was restored to control levels. Leaving K^+^-outward currents and Na^+^-inward currents unaltered dexamethasone selectively prevented the downregulation of the inward K^+^-current in the majority of the precursor cells investigated, lending support to the speculation, that the block of the down-regulation of the inwardly rectifying K^+ ^current could play an essential part in the protective role of dexamethasone. In line with the concept, that the reduction of the inwardly rectifying K^+^-current plays a causal role in oligodendrocyte damage, in the lethal Kir4.1 knockout mouse, depolarized membrane potentials of oligodendrocytes as well as a disturbed myelination with vacuolized white matter are found [54]. Thus, the following conception emerges: Cytokine treatment of oligodendrocyte precursor cells causes a down-regulation of inwardly rectifying potassium currents. The resulting depolarisation could trigger a concerted programme leading to cell death and possibly block of differentiation of the surviving cells.

### Clinical implications

Corticosteroids are widely used in perinatal medicine to induce lung maturation. Although our results indicate a clear protective effect of corticosteroids, especially dexamethasone, on oligodendrocyte precursor cells, studies examining the effects of corticosteroid treatment on preterm infants do not indicate umambiguously protective effects [55] and in some studies even adverse effects on the development of PVL [56] have been decribed. Although the adverse effects of intraveneously administered dexamethasone could have resulted from sulphites in the injectable preparation [57], additional deleterious effects of dexamethasone on brain development [58] and hippocampal neurons [59] have been reported. Hence it remains to be investigated, whether other steroids, such as methylprednisolone or betamethasone, which have been shown to induce less side effects [56], exert a comparable protective effect on oligodendrocyte precursor cells or whether other agents interfering with the pathway leading to a down-regulation of inwardly rectifying K^+ ^currents could be employed as protectants.

## Conclusion

Our results indicate, that cytokine-induced damage to oligodendrocyte precursor cells can be prevented at the level of morphological maturation and the expression of mature myelin proteins by corticosteroids. Furthermore, even the physiological function of the damaged cells, as reflected by the restoration of the differentiated pattern of the voltage-activated ion currents was rescued. This indicates that cytokine-damaged immature oligodendrocytes could eventually be functionally restituted by an adequate pharmacological treatment. Since clinical studies so far provided less clear results using corticosteroids more detailed investigations concerning alternative approaches to interfere with the molecular pathways activated by cytokines in oligodendrocyte precursor cells have to be worked out in order to find clinically more successful agents to protect immature oligodendrocyte precursors.

## Competing interests

The authors declare that they have no competing interests.

## Authors' contributions

SICM measurements were performed by SAM, who also performed the majority of the whole cell current recordings and data evaluation, assisted by SS, who also prepared part of the cultures. RM performed additional patch clamp recordings, especially of dexamethasone co-treated cells at day 3 in vitro. Western blot, cell culture and immunocytochemistry were performed mainly by BV. RB, RH and IDD conceived the project and study, participated in its execution and coordination and prepared the manuscript with the assistance of the other authors who read and approved the resultant manuscript.

## References

[B1] Dammann O, Leviton A (1997). Maternal intrauterine infection, cytokines, and brain damage in the preterm newborn. Pediatr Res.

[B2] Berger R, Garnier Y (1999). Pathophysiology of perinatal brain damage. Brain Res Brain Res Rev.

[B3] Ellison VJ, Mocatta TJ, Winterbourn CC, Darlow BA, Volpe JJ, Inder TE (2005). The relationship of CSF and plasma cytokine levels to cerebral white matter injury in the premature newborn. Pediatr Res.

[B4] du Plessis AJ, Volpe JJ (2002). Perinatal brain injury in the preterm and term newborn. Curr Opin Neurol.

[B5] Mesples B, Plaisant F, Fontaine RH, Gressens P (2005). Pathophysiology of neonatal brain lesions: lessons from animal models of excitotoxicity. Acta Paediatr.

[B6] Folkerth RD, Keefe RJ, Haynes RL, Trachtenberg FL, Volpe JJ, Kinney HC (2004). Interferon-gamma expression in periventricular leukomalacia in the human brain. Brain Pathol.

[B7] Agresti C, D'Urso D, Levi G (1996). Reversible inhibitory effects of interferon-gamma and tumour necrosis factor-alpha on oligodendroglial lineage cell proliferation and differentiation in vitro. Eur J Neurosci.

[B8] Cammer W, Zhang H (1999). Maturation of oligodendrocytes is more sensitive to TNF alpha than is survival of precursors and immature oligodendrocytes. J Neuroimmunol.

[B9] Andrews T, Zhang P, Bhat NR (1998). TNFalpha potentiates IFNgamma-induced cell death in oligodendrocyte progenitors. J Neurosci Res.

[B10] Feldhaus B, Dietzel ID, Heumann R, Berger R (2004). Effects of interferon-γ and tumor necrosis factor-α on survival and differentiation of oligodendrocyte progenitors. J Soc Gynecol Investig.

[B11] Chew LJ, King WC, Kennedy A, Gallo V (2005). Interferon-gamma inhibits cell cycle exit in differentiating oligodendrocyte progenitor cells. Glia.

[B12] Sontheimer H, Trotter J, Schachner M, Kettenmann H (1989). Channel expression correlates with differentiation stage during the development of oligodendrocytes from their precursor cells in culture. Neuron.

[B13] Barres BA, Koroshetz WJ, Swartz KJ, Chun LL, Corey DP (1990). Ion channel expression by white matter glia: the O-2A glial progenitor cell. Neuron.

[B14] Barres BA, Chun LL, Corey DP (1988). Ion channel expression by white matter glia: I. Type 2 astrocytes and oligodendrocytes. Glia.

[B15] Chiu SY, Wilson GF (1989). The role of potassium channels in Schwann cell proliferation in Wallerian degeneration of explant rabbit sciatic nerves. J Physiol (Lond).

[B16] Soliven B, Ma L, Bae H, Attali B, Sobko A, Iwase T (2003). PDGF upregulates delayed rectifier via Src family kinases and sphingosine kinase in oligodendroglial progenitors. Am J Physiol Cell Physiol.

[B17] MacFarlane SN, Sontheimer H (2000). Changes in ion channel expression accompany cell cycle progression of spinal cord astrocytes. Glia.

[B18] Vautier F, Belachew S, Chittajallu R, Gallo V (2004). Shaker-type potassium channel subunits differentially control oligodendrocyte progenitor proliferation. Glia.

[B19] Kofuji P, Newman EA (2004). Potassium buffering in the central nervous system. Neurosci.

[B20] La Mantia L, Eoli M, Milanese C, Salmaggi A, Dufour A, Torri V (1994). Double-blind trial of dexamethasone versus methylprednisolone in multiple sclerosis acute relapses. Eur Neurol.

[B21] Durelli L, Cocito D, Riccio A, Barile C, Bergamasco B, Baggio GF, Perla F, Delsedime M, Gusmaroli G, Bergamini L (1986). High-dose intravenous methylprednisolone in the treatment of multiple sclerosis: clinical-immunologic correlations. Neurology.

[B22] Melcangi RC, Cavarretta I, Magnaghi V, Ciusani E, Salmaggi A (2000). Corticosteroids protect oligodendrocytes from cytokine-induced cell death. Neuroreport.

[B23] McCarthy KD, de Vellis J (1980). Preparation of separate astroglial and oligodendroglial cell cultures from rat cerebral tissue. J Cell Biol.

[B24] Back SA, Gan X, Li Y, Rosenberg PA, Volpe JJ (1998). Maturation-dependent vulnerability of oligodendrocytes to oxidative stress-induced death caused by glutathione depletion. J Neurosci.

[B25] Hansma PK, Drake B, Marti O, Gould SAC, Prater CB (1989). The scanning ion-conductance microscope. Science.

[B26] Korchev YE, Gorelik J, Lab MJ, Sviderskaya EV, Johnston CL, Coombes CR, Vodyanoy I, Edwards CRW (2000). Cell volume measurement using scanning ion conductance microscopy. Biophys J.

[B27] Mann SA, Hoffmann G, Hengstenberg A, Schuhmann W, Dietzel ID (2002). Pulse-mode scanning ion conductance microscopy-a method to investigate cultured hippocampal cells. J Neurosci Methods.

[B28] Happel P, Hoffmann G, Mann SA, Dietzel ID (2003). Monitoring cell movements and volume changes with pulse-mode scanning ion conductance microscopy. J Microsc.

[B29] Mann SA, Meyer JW, Dietzel ID (2006). Integration of a scanning ion conductance microscope into phase-contrast optics and its application to the quantification of morphological parameters of selected cells. J Microscopy.

[B30] Ransom CB, Sontheimer H (1995). Biophysical and pharmacological characterization of inwardly rectifying K^+ ^currents in rat spinal cord astrocytes. J Neurophysiol.

[B31] Feldhaus B, Dietzel ID, Heumann R, Berger R (2004). Kortikoide schützen Oligodendrozyten-Vorläuferzellen vor Zytokin-induzierten Schäden. Zentralbl Gynakol.

[B32] Sakmann B, Trube G (1984). Voltage-dependent inactivation of inward-rectifying single-channel currents in the guinea-pig heart cell membrane. J Physiol.

[B33] Williamson AV, Compston DA, Randall AD (1997). Analysis of the ion channel complement of the rat oligodendrocyte progenitor in a commonly studied in vitro preparation. Eur J Neurosci.

[B34] Molina-Holgado E, Vela JM, Arevalo-Martin A, Almazan G, Molina-Holgado F, Borrell J, Guaza C (2002). Cannabinoids promote oligodendrocyte progenitor survival: involvement of cannabinoid receptors and phosphatidylinositol-3 kinase/Akt signaling. J Neurosci.

[B35] Barres BA, Lazar MA, Raff MC (1994). A novel role for thyroid hormone, glucocorticoids and retinoic acid in timing oligodendrocyte development. Development.

[B36] Alonso G (2000). Prolonged corticosterone treatment of adult rats inhibits the proliferation of oligodendrocyte progenitors present throughout white and gray matter regions of the brain. Glia.

[B37] Kumar S, Cole R, Chiappelli F, de Vellis J (1989). Differential regulation of oligodendrocyte markers by glucocorticoids: Post-transcriptional regulation of both proteolipid protein and myelin basic protein and transcriptional regulation of glycerol phosphate dehydrogenase. Proc Natl Acad Sci USA.

[B38] Cheng JD, de Vellis J (2000). Oligodendrocytes as glucocorticoids target cells: functional analysis of the glycerol phosphate dehydrogenase gene. J Neurosci Res.

[B39] Poduslo SE, Pak CH, Miller K (1990). Hydrocortisone induction during oligodendroglial differentiation. Neurosci Lett.

[B40] Ved HS, Gustow E, Pieringer RA (1989). Effect of hydrocortisone on myelin basic protein in developing primary brain cultures. Neurosci Lett.

[B41] Byravan S, Campagnoni AT (1994). Serum factors and hydrocortisone influence the synthesis of myelin basic proteins in mouse brain primary cultures. Int J Dev Neurosci.

[B42] Melcangi RC, Magnaghi V, Cavarretta I, Riva MA, Martini L (1997). Corticosteroid effects on gene expression of myelin basic protein in oligodendrocytes and of glial fibrillary acidic protein in type 1 astrocytes. J Neuroendocrinol.

[B43] Almazan G, Honegger P, Du PP, Matthieu JM (1986). Dexamethasone stimulates the biochemical differentiation of fetal forebrain cells in reaggregating cultures. Dev Neurosci.

[B44] Gorman AM, Hirt UA, Orrenius S, Ceccatelli S (2000). Dexamethasone pre-treatment interferes with apoptotic death in glioma cells. Neurosci.

[B45] Pagliacci MC, Migliorati G, Smacchia M, Grignani F, Riccardi C, Nicoletti I (1993). Cellular stress and glucocorticoid hormones protect L929 mouse fibroblasts from tumor necrosis factor alpha cytotoxicity. J Endocrinol Invest.

[B46] Evans-Storms RB, Cidlowski JA (2000). Delineation of an antiapoptotic action of glucocorticoids in hepatoma cells: the role of nuclear factor-kappaB. Endocrinol.

[B47] Planey SL, Litwack G (2000). Glucocorticoid-induced apoptosis in lymphocytes. Biochem Biophys Res Commun.

[B48] Smets LA, Salomons G, Berg J van den (1999). Glucocorticoid induced apoptosis in leukemia. Adv Exp Med Biol.

[B49] Lampert A, Muller MM, Berchtold S, Lang KS, Palmada M, Dobrovinskaya O, Lang F (2003). Effect of dexamethasone on voltage-gated K^+ ^channels in Jurkat T-lymphocytes. Pflugers Arch.

[B50] Takimoto K, Fomina AF, Gealy R, Trimmer JS, Levitan ES (1993). Dexamethasone rapidly induces Kv1.5 K^+ ^channel gene transcription and expression in clonal pituitary cells. Neuron.

[B51] Huang MH, So EC, Liu YC, Wu SN (2006). Glucocorticoids stimulate the activity of large-conductance Ca^2+^-activated K^+ ^channels in pituitary GH3 and AtT-20 cells via a non-genomic mechanism. Steroids.

[B52] Soliven B, Szuchet S, Nelson DJ (1991). Tumor necrosis factor inhibits K^+ ^current expression in cultured oligodendrocytes. J Membr Biol.

[B53] McLarnon JG, Michikawa M, Kim SU (1993). Effects of tumor necrosis factor on inward potassium current and cell morphology in cultured human oligodendrocytes. Glia.

[B54] Neusch C, Rozengurt N, Jacobs RE, Lester HA, Kofuji P (2001). Kir4.1 potassium channel subunit is crucial for oligodendrocyte development and in vivo myelination. J Neurosci.

[B55] Smrcek JM, Schwartau N, Kohl M, Berg C, Geipel A, Krapp M, Diedrich K, Ludwig M (2005). Antenatal corticosteroid therapy in premature infants. Arch Gynecol Obstet.

[B56] Spinillo A, Viazzo F, Colleoni R, Chiara A, Maria CR, Fazzi E (2004). Two-year infant neurodevelopmental outcome after single or multiple antenatal courses of corticosteroids to prevent complications of prematurity. Am J Obstet Gynecol.

[B57] Baud O (2004). Postnatal steroid treatment and brain development. Arch Dis Child Fetal Neonatal Ed.

[B58] Matthews SG (2000). Antenatal glucocorticoids and programming of the developing CNS. Pediatr Res.

[B59] Crochemore C, Lu J, Wu Y, Liposits Z, Sousa N, Holsboer F, Almeida OF (2005). Direct targeting of hippocampal neurons for apoptosis by glucocorticoids is reversible by mineralocorticoid receptor activation. Mol Psychiatry.

